# 4-Methyl-*N*-[2-(2-phenyl­ethyn­yl)phen­yl]-*N*-(prop-2-yn-1-yl)benzene-1-sulfonamide

**DOI:** 10.1107/S2414314625009435

**Published:** 2025-10-31

**Authors:** Benjamin Dassonneville, Heiner Detert, Dieter Schollmeyer

**Affiliations:** a University of Mainz, Department of Chemistry, Duesbergweg 10-14, 55099 Mainz, Germany; b University Mainz, Duesbergweg 10-14, 55099 Mainz, Germany; Katholieke Universiteit Leuven, Belgium

**Keywords:** crystal structure, hydrogen bridge, alkyne, sulfonamide

## Abstract

The title compound, C_24_H_19_NO_2_S, was prepared by propargylation of the sulfonamide. The tolyl group points to the planar tolane unit. In the extended structure, two inter­molecular hydrogen bridges connect the mol­ecules to form chains.

## Graphical abstract

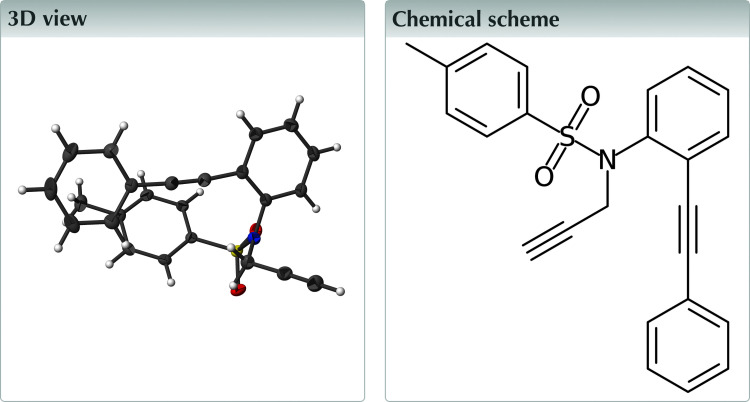

## Structure description

**Figure 1:**
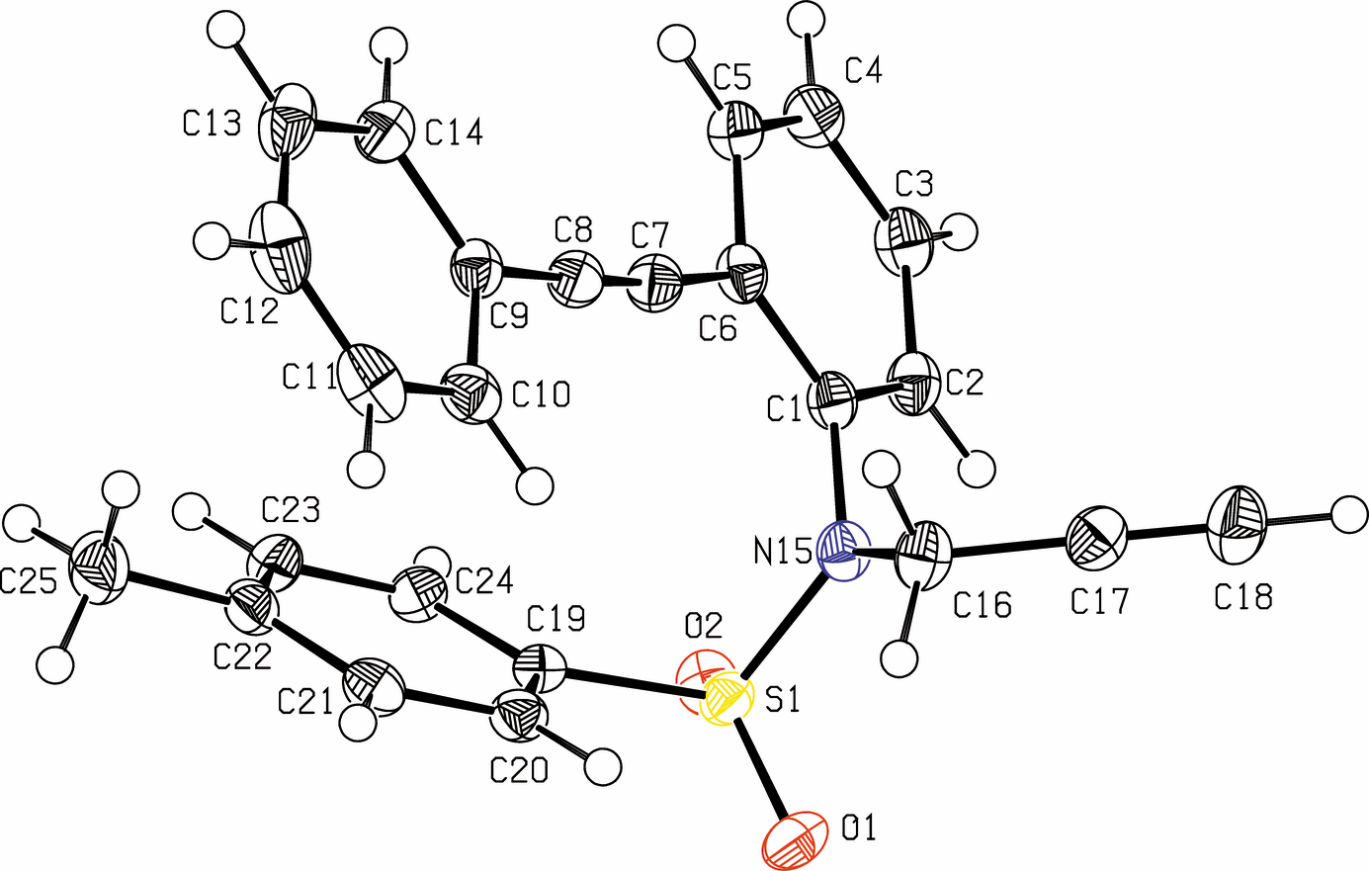
View (Spek, 2009 ▸) of the title compound. Displacement ellipsoids are drawn at the 50% probability level.

**Figure 2:**
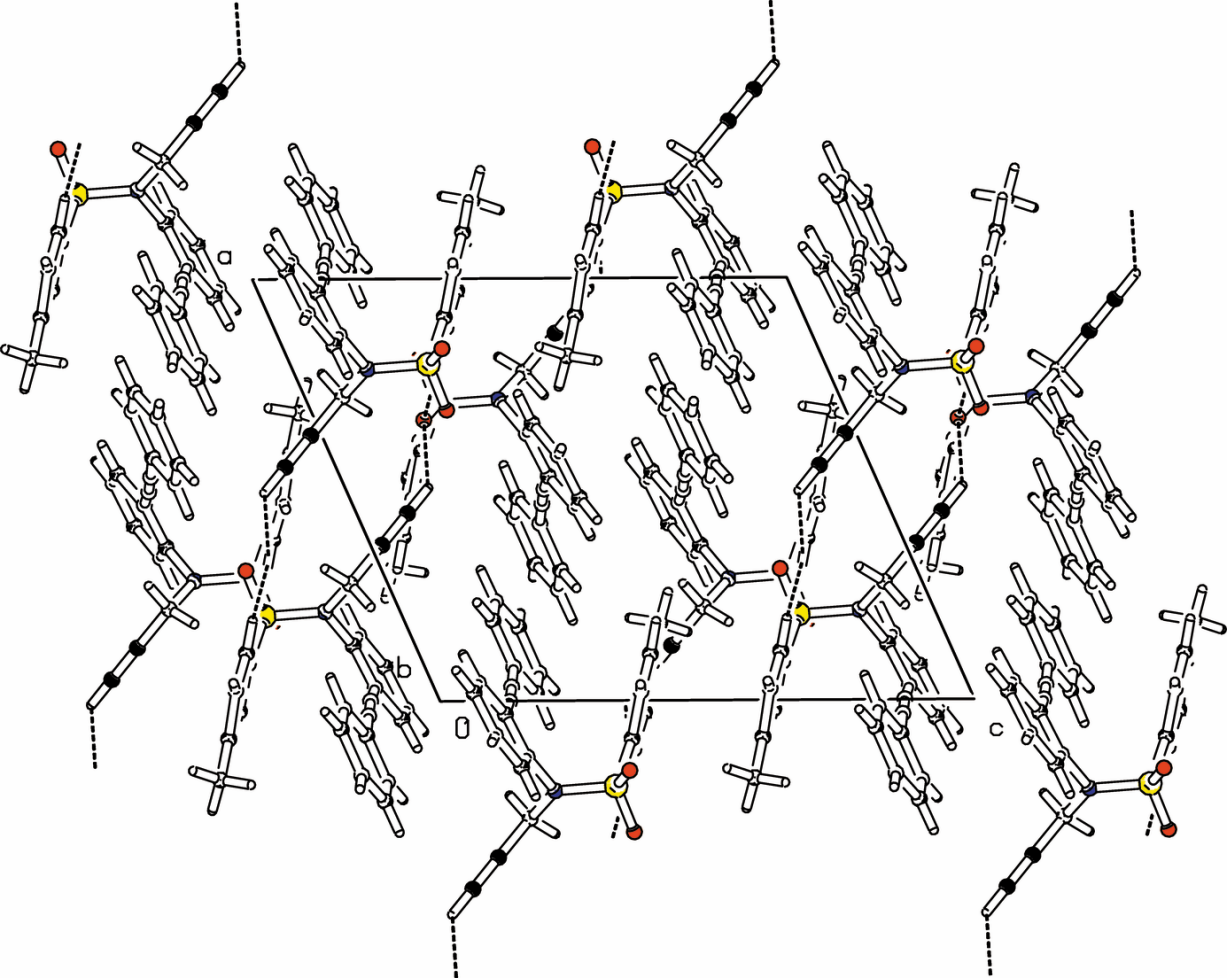
Part of the packing diagram. View along *b*-axis direction (Spek, 2009 ▸).

The title compound, C_24_H_19_NO_2_S (Fig. 1 ▸), was prepared as part of a larger project on the synthesis of condensed heterocycles *via* transition metal catalysis (Dassonneville *et al.*, 2011 ▸; Letessier *et al.*, 2012 ▸, 2013 ▸). Whereas *O,N*-dialkynyl sulfonyl­anilines were successfully converted to carbolines or indolo­thio­pyranes (Dassonneville *et al.*, 2023*a* ▸,*b* ▸), attempts to cyclize homologous *N*-propargyl derivatives failed. The title compound forms monoclinic crystals with four mol­ecules per unit cell. The C1–C14 tolane unit is close to planar [dihedral angle between the phenyl rings: 5.48 (7)°] with very similar propargylic bonds C6—C7 [1.4303 (19) Å] and C8—C9 [1.4328 (19) Å]. The tolyl group points in the direction of the tolane with dihedral angle between the tolyl unit and the terminal phenyl (C9—C14) ring of 38.37 (7)°. The alkyne points towards the benzene (C1–C6) ring of the tolane, as reflected in the torsion angle of 124.65 (12)° for the C17—C16—N15—S1 grouping. In the crystal, two C—H⋯O hydrogen bonds (Table 1 ▸) connect the mol­ecules to form chains. Neighbouring chains are connected via overlapping tolane units to form layers parallel to (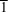
101). The C20—H20⋯O2 bridge is characterized by a C⋯O distance of 3.3110 (18) Å (O2⋯H20: 2.44 Å) and a C—H⋯O angle of 152°. The other bridge along C18—H18⋯O2 has a length of C18⋯O2 = 3.231 (2) Å (H18⋯O2: 2.45 Å) with a C—H⋯O angle of 139°. Within the chain, every second mol­ecule is related *via* a twofold screw axis whereas the mol­ecules in between are symmetrically related *via* a glide plane (Fig. 2 ▸).

## Synthesis and crystallization

The synthesis of the title compound was performed by deprotonation of *N*-tosyl-2-phenyl­ethynylaniline (Amjad *et al.* 2004 ▸; Martínez-Esperón *et al.*, 2008 ▸) with potassium hexamethyldisilazanide (KHMDS) at low (*ca* 200 K) temperature and reaction with progargylic bromide. Purification by column chromatography on silica with toluene as eluent gave the title compound (*R*_f_ = 0.23) and a by-product. The title compound crystallized from toluene as off-white cubes with m.p. = 381.5 K. The assignment of NMR signals is based on two-dimensional spectra and follows IUPAC nomenclature. ^1^H-NMR (CDCl_3_, 600 MHz): 7.71 (*d*, *J* = 7.9 Hz, 2 H, 3-H, 5-H, tol), 7.56 (*m*, 1 H), 7.46 (*m*, 1 H), 7.37 (*m*, 4 H), 7.33 (*m*, 3 H), 7.16 (*d*, 2 H, *J* = 7.9 Hz, 2-H, 6-H, tol), 4.65 (*bs*, 2 H, 1-H prop), 2.28 (*s*, 3 H, CH_3_), 2.24 (*t*, 1 H, *J* = 2.5 Hz, 3-H prop); ^13^C-NMR (CDCl_3_ 600 MHz): 143.6 (C1, tol), 139.5, 137.1, 133.4 (^1^*J*_CH_ = 47 Hz), 132.1, 131.6 (2 C), 129.6 (2 C), 129.0, 128.7, 128.7, 128.3 (2 C), 127.7 (2 C), 123.8, 122.7, 94.5, 85.8, 78.3, 73.5 (^1^*J*_CH_ = 210 Hz, C-3 prop), 40.2 (C-1, prop), 21.5 (CH_3_).

## Refinement

Crystal data, data collection and structure refinement details are summarized in Table 2 ▸.

## Supplementary Material

Crystal structure: contains datablock(s) I, global. DOI: 10.1107/S2414314625009435/vm4074sup1.cif

Structure factors: contains datablock(s) I. DOI: 10.1107/S2414314625009435/vm4074Isup2.hkl

Supporting information file. DOI: 10.1107/S2414314625009435/vm4074Isup3.cml

CCDC reference: 2498200

Additional supporting information:  crystallographic information; 3D view; checkCIF report

## Figures and Tables

**Table 1:** Hydrogen-bond geometry (Å, °)

*D*—H⋯*A*	*D*—H	H⋯*A*	*D*⋯*A*	*D*—H⋯*A*
C20—H20⋯O2^i^	0.95	2.44	3.3110 (18)	152
C18—H18⋯O2^ii^	0.95	2.45	3.231 (2)	139

Symmetry codes: (i) 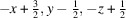; (ii) 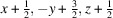.

**Table 2:** Experimental details

Crystal data	
Chemical formula	C_24_H_19_NO_2_S
*M*_r_	385.46
Crystal system, space group	Monoclinic, *P*2_1_/*n*
Temperature (K)	120
*a*, *b*, *c* (Å)	12.4866 (5), 11.7479 (3), 14.4481 (6)
β (°)	113.728 (3)
*V* (Å^3^)	1940.25 (13)
*Z*	4
Radiation type	Mo *K*α
μ (mm^−1^)	0.19
Crystal size (mm)	0.45 × 0.37 × 0.27

Data collection	
Diffractometer	Stoe IPDS 2T
Absorption correction	Integration (Stoe & Cie, 2020 ▸)
*T*_min_, *T*_max_	0.919, 0.962
No. of measured, independent and observed [*I* > 2σ(*I*)] reflections	9492, 4603, 4075
*R*_int_	0.022
(sin θ/λ)_max_ (Å^−1^)	0.657

Refinement	
*R*[*F*^2^ > 2σ(*F*^2^)], *wR*(*F*^2^), *S*	0.039, 0.101, 1.06
No. of reflections	4603
No. of parameters	254
H-atom treatment	H-atom parameters constrained
Δρ_max_, Δρ_min_ (e Å^−3^)	0.35, −0.36

Computer programs: *X-AREAWinXpose*, *Recipe* and *Integrate* (Stoe & Cie, 2020 ▸), *SHELXT2014* (Sheldrick, 2015*a* ▸), *SHELXL2019/2* (Sheldrick, 2015*b* ▸) and *PLATON* (Spek, 2009 ▸).

## full crystallographic data

### 4-Methyl-N-[2-(2-phenylethynyl)phenyl]-N-(prop-2-yn-1-yl)benzene-\ 1-sulfonamide Crystal data

**Table:**

C_24_H_19_NO_2_S	*D*_x_ = 1.320 Mg m^−^^3^
*M**_r_* = 385.46	Melting point: 381.5 K
Monoclinic, *P*2_1_/*n*	Mo *K*α radiation, λ = 0.71073 Å
*a* = 12.4866 (5) Å	Cell parameters from 13782 reflections
*b* = 11.7479 (3) Å	θ = 2.5–28.4°
*c* = 14.4481 (6) Å	µ = 0.19 mm^−^^1^
β = 113.728 (3)°	*T* = 120 K
*V* = 1940.25 (13) Å^3^	Block, colorless
*Z* = 4	0.45 × 0.37 × 0.27 mm
*F*(000) = 808	

### 4-Methyl-N-[2-(2-phenylethynyl)phenyl]-N-(prop-2-yn-1-yl)benzene-\ 1-sulfonamide Data collection

**Table:**

Stoe IPDS 2T diffractometer	4603 independent reflections
Radiation source: sealed X-ray tube, 12 x 0.4 mm long-fine focus	4075 reflections with *I* > 2σ(*I*)
Detector resolution: 6.67 pixels mm^-1^	*R*_int_ = 0.022
rotation method, ω scans	θ_max_ = 27.9°, θ_min_ = 2.5°
Absorption correction: integration software?; reference?	*h* = −16→16
*T*_min_ = 0.919, *T*_max_ = 0.962	*k* = −15→13
9492 measured reflections	*l* = −18→18

### 4-Methyl-N-[2-(2-phenylethynyl)phenyl]-N-(prop-2-yn-1-yl)benzene-\ 1-sulfonamide Refinement

**Table:**

Refinement on *F*^2^	Primary atom site location: dual
Least-squares matrix: full	Hydrogen site location: inferred from neighbouring sites
*R*[*F*^2^ > 2σ(*F*^2^)] = 0.039	H-atom parameters constrained
*wR*(*F*^2^) = 0.101	*w* = 1/[σ^2^(*F*_o_^2^) + (0.0466*P*)^2^ + 0.9953*P*] where *P* = (*F*_o_^2^ + 2*F*_c_^2^)/3
*S* = 1.06	(Δ/σ)_max_ = 0.001
4603 reflections	Δρ_max_ = 0.35 e Å^−^^3^
254 parameters	Δρ_min_ = −0.36 e Å^−^^3^
0 restraints	

### 4-Methyl-N-[2-(2-phenylethynyl)phenyl]-N-(prop-2-yn-1-yl)benzene-\ 1-sulfonamide Special details

**Table:**

Geometry. All esds (except the esd in the dihedral angle between two l.s. planes) are estimated using the full covariance matrix. The cell esds are taken into account individually in the estimation of esds in distances, angles and torsion angles; correlations between esds in cell parameters are only used when they are defined by crystal symmetry. An approximate (isotropic) treatment of cell esds is used for estimating esds involving l.s. planes.
Refinement. Hydrogen atoms attached to carbons were placed at calculated positions and were refined in the riding-model approximation with C–H = 0.99 Å, and with *U*_iso_(H) = 1.2 *U*_eq_(C).

### 4-Methyl-N-[2-(2-phenylethynyl)phenyl]-N-(prop-2-yn-1-yl)benzene-\ 1-sulfonamide Fractional atomic coordinates and isotropic or equivalent isotropic displacement parameters (Å^2^)

**Table:**

	*x*	*y*	*z*	*U*_iso_*/*U*_eq_	
S1	0.70257 (3)	0.61385 (3)	0.24207 (2)	0.02229 (10)	
O1	0.81074 (9)	0.56893 (10)	0.24567 (9)	0.0329 (3)	
O2	0.66567 (9)	0.72594 (9)	0.20338 (7)	0.0276 (2)	
C1	0.63253 (11)	0.68233 (11)	0.38380 (9)	0.0210 (3)	
C2	0.65685 (12)	0.79737 (12)	0.40293 (10)	0.0245 (3)	
H2	0.726052	0.828453	0.400793	0.029*	
C3	0.58094 (13)	0.86706 (12)	0.42508 (11)	0.0275 (3)	
H3	0.597557	0.945912	0.437140	0.033*	
C4	0.48040 (13)	0.82170 (13)	0.42969 (11)	0.0281 (3)	
H4	0.427802	0.869627	0.444157	0.034*	
C5	0.45715 (12)	0.70698 (13)	0.41321 (10)	0.0260 (3)	
H5	0.389315	0.676214	0.418217	0.031*	
C6	0.53175 (11)	0.63495 (12)	0.38922 (10)	0.0220 (3)	
C7	0.50115 (12)	0.51736 (12)	0.37016 (10)	0.0239 (3)	
C8	0.46657 (12)	0.42115 (12)	0.35438 (10)	0.0236 (3)	
C9	0.41978 (11)	0.30811 (12)	0.33417 (10)	0.0218 (3)	
C10	0.48218 (13)	0.21974 (13)	0.31371 (10)	0.0265 (3)	
H10	0.557670	0.233824	0.314953	0.032*	
C11	0.43428 (16)	0.11233 (13)	0.29175 (11)	0.0340 (3)	
H11	0.476185	0.052954	0.276443	0.041*	
C12	0.32517 (16)	0.09043 (14)	0.29188 (12)	0.0377 (4)	
H12	0.292628	0.016165	0.276887	0.045*	
C13	0.26402 (14)	0.17648 (14)	0.31377 (12)	0.0337 (3)	
H13	0.189803	0.160897	0.314707	0.040*	
C14	0.30993 (12)	0.28574 (13)	0.33447 (10)	0.0261 (3)	
H14	0.266990	0.344925	0.348750	0.031*	
N15	0.71296 (10)	0.61439 (10)	0.35906 (9)	0.0230 (2)	
C16	0.77863 (12)	0.52334 (12)	0.42943 (11)	0.0267 (3)	
H16A	0.724042	0.477366	0.448299	0.032*	
H16B	0.814650	0.472514	0.395324	0.032*	
C17	0.87018 (12)	0.57058 (13)	0.52094 (11)	0.0276 (3)	
C18	0.94493 (14)	0.60672 (14)	0.59514 (13)	0.0355 (4)	
H18	1.005039	0.635792	0.654812	0.043*	
C19	0.59043 (11)	0.51880 (11)	0.17274 (9)	0.0203 (3)	
C20	0.61574 (12)	0.40336 (12)	0.17825 (10)	0.0231 (3)	
H20	0.692954	0.377034	0.216646	0.028*	
C21	0.52737 (13)	0.32719 (12)	0.12728 (10)	0.0251 (3)	
H21	0.544846	0.248336	0.128784	0.030*	
C22	0.41271 (12)	0.36439 (12)	0.07357 (10)	0.0242 (3)	
C23	0.39009 (12)	0.48050 (13)	0.06791 (10)	0.0252 (3)	
H23	0.312802	0.506968	0.030008	0.030*	
C24	0.47833 (11)	0.55862 (12)	0.11664 (10)	0.0227 (3)	
H24	0.462188	0.637945	0.111623	0.027*	
C25	0.31639 (14)	0.27906 (15)	0.02433 (12)	0.0350 (3)	
H25A	0.243835	0.319163	−0.016472	0.052*	
H25B	0.304597	0.234149	0.076625	0.052*	
H25C	0.338064	0.228357	−0.019186	0.052*	

### 4-Methyl-N-[2-(2-phenylethynyl)phenyl]-N-(prop-2-yn-1-yl)benzene-\ 1-sulfonamide Atomic displacement parameters (Å^2^)

**Table:**

	*U*^11^	*U*^22^	*U*^33^	*U*^12^	*U*^13^	*U*^23^
S1	0.01870 (16)	0.02383 (18)	0.02494 (17)	−0.00220 (12)	0.00939 (12)	−0.00065 (12)
O1	0.0205 (5)	0.0407 (6)	0.0402 (6)	−0.0017 (4)	0.0148 (4)	−0.0037 (5)
O2	0.0314 (5)	0.0234 (5)	0.0276 (5)	−0.0050 (4)	0.0115 (4)	0.0023 (4)
C1	0.0215 (6)	0.0200 (6)	0.0183 (6)	0.0010 (5)	0.0048 (5)	0.0007 (5)
C2	0.0245 (6)	0.0225 (7)	0.0237 (6)	−0.0034 (5)	0.0068 (5)	0.0000 (5)
C3	0.0314 (7)	0.0201 (7)	0.0270 (7)	0.0000 (5)	0.0075 (6)	−0.0020 (5)
C4	0.0278 (7)	0.0288 (7)	0.0251 (7)	0.0057 (6)	0.0081 (5)	−0.0013 (5)
C5	0.0237 (6)	0.0300 (7)	0.0229 (6)	−0.0005 (5)	0.0080 (5)	−0.0003 (5)
C6	0.0232 (6)	0.0221 (6)	0.0180 (6)	−0.0015 (5)	0.0054 (5)	0.0010 (5)
C7	0.0233 (6)	0.0254 (7)	0.0228 (6)	−0.0017 (5)	0.0090 (5)	0.0014 (5)
C8	0.0236 (6)	0.0251 (7)	0.0220 (6)	−0.0010 (5)	0.0091 (5)	0.0020 (5)
C9	0.0233 (6)	0.0219 (6)	0.0182 (6)	−0.0014 (5)	0.0061 (5)	0.0028 (5)
C10	0.0295 (7)	0.0280 (7)	0.0214 (6)	0.0031 (6)	0.0098 (5)	0.0029 (5)
C11	0.0489 (9)	0.0230 (7)	0.0254 (7)	0.0052 (6)	0.0103 (6)	0.0004 (5)
C12	0.0508 (10)	0.0233 (7)	0.0283 (8)	−0.0109 (7)	0.0045 (7)	0.0011 (6)
C13	0.0286 (7)	0.0370 (9)	0.0290 (7)	−0.0113 (6)	0.0049 (6)	0.0081 (6)
C14	0.0242 (7)	0.0274 (7)	0.0254 (7)	−0.0003 (5)	0.0087 (5)	0.0053 (5)
N15	0.0223 (5)	0.0217 (6)	0.0222 (5)	0.0025 (4)	0.0061 (4)	0.0009 (4)
C16	0.0243 (7)	0.0221 (7)	0.0284 (7)	0.0024 (5)	0.0051 (5)	0.0026 (5)
C17	0.0245 (7)	0.0270 (7)	0.0291 (7)	0.0041 (6)	0.0085 (6)	0.0043 (6)
C18	0.0280 (7)	0.0355 (9)	0.0341 (8)	0.0029 (6)	0.0033 (6)	−0.0002 (6)
C19	0.0198 (6)	0.0226 (6)	0.0202 (6)	−0.0004 (5)	0.0099 (5)	−0.0010 (5)
C20	0.0227 (6)	0.0247 (7)	0.0226 (6)	0.0045 (5)	0.0100 (5)	0.0004 (5)
C21	0.0316 (7)	0.0206 (6)	0.0248 (6)	0.0019 (5)	0.0131 (6)	−0.0013 (5)
C22	0.0266 (7)	0.0275 (7)	0.0193 (6)	−0.0043 (5)	0.0100 (5)	−0.0016 (5)
C23	0.0195 (6)	0.0296 (7)	0.0249 (6)	0.0017 (5)	0.0073 (5)	0.0007 (5)
C24	0.0222 (6)	0.0211 (6)	0.0252 (6)	0.0024 (5)	0.0098 (5)	0.0012 (5)
C25	0.0345 (8)	0.0357 (9)	0.0324 (8)	−0.0112 (7)	0.0110 (6)	−0.0062 (6)

### 4-Methyl-N-[2-(2-phenylethynyl)phenyl]-N-(prop-2-yn-1-yl)benzene-\ 1-sulfonamide Geometric parameters (Å, º)

**Table:**

S1—O1	1.4314 (11)	C12—H12	0.9500
S1—O2	1.4324 (11)	C13—C14	1.388 (2)
S1—N15	1.6424 (12)	C13—H13	0.9500
S1—C19	1.7556 (13)	C14—H14	0.9500
C1—C2	1.3883 (19)	N15—C16	1.4763 (17)
C1—C6	1.4067 (18)	C16—C17	1.465 (2)
C1—N15	1.4362 (17)	C16—H16A	0.9900
C2—C3	1.384 (2)	C16—H16B	0.9900
C2—H2	0.9500	C17—C18	1.181 (2)
C3—C4	1.390 (2)	C18—H18	0.9500
C3—H3	0.9500	C19—C24	1.3864 (18)
C4—C5	1.379 (2)	C19—C20	1.3875 (19)
C4—H4	0.9500	C20—C21	1.381 (2)
C5—C6	1.4013 (19)	C20—H20	0.9500
C5—H5	0.9500	C21—C22	1.396 (2)
C6—C7	1.4303 (19)	C21—H21	0.9500
C7—C8	1.199 (2)	C22—C23	1.389 (2)
C8—C9	1.4328 (19)	C22—C25	1.506 (2)
C9—C14	1.3983 (19)	C23—C24	1.3884 (19)
C9—C10	1.399 (2)	C23—H23	0.9500
C10—C11	1.378 (2)	C24—H24	0.9500
C10—H10	0.9500	C25—H25A	0.9800
C11—C12	1.387 (3)	C25—H25B	0.9800
C11—H11	0.9500	C25—H25C	0.9800
C12—C13	1.378 (3)		
			
O1—S1—O2	120.25 (7)	C14—C13—H13	119.7
O1—S1—N15	106.34 (6)	C13—C14—C9	119.58 (14)
O2—S1—N15	106.13 (6)	C13—C14—H14	120.2
O1—S1—C19	108.09 (6)	C9—C14—H14	120.2
O2—S1—C19	107.74 (6)	C1—N15—C16	118.78 (11)
N15—S1—C19	107.71 (6)	C1—N15—S1	119.00 (9)
C2—C1—C6	120.20 (13)	C16—N15—S1	119.87 (10)
C2—C1—N15	118.14 (12)	C17—C16—N15	111.26 (12)
C6—C1—N15	121.66 (12)	C17—C16—H16A	109.4
C3—C2—C1	120.44 (13)	N15—C16—H16A	109.4
C3—C2—H2	119.8	C17—C16—H16B	109.4
C1—C2—H2	119.8	N15—C16—H16B	109.4
C2—C3—C4	120.01 (13)	H16A—C16—H16B	108.0
C2—C3—H3	120.0	C18—C17—C16	178.78 (17)
C4—C3—H3	120.0	C17—C18—H18	180.0
C5—C4—C3	119.86 (13)	C24—C19—C20	121.10 (12)
C5—C4—H4	120.1	C24—C19—S1	120.31 (10)
C3—C4—H4	120.1	C20—C19—S1	118.55 (10)
C4—C5—C6	121.21 (13)	C21—C20—C19	119.21 (13)
C4—C5—H5	119.4	C21—C20—H20	120.4
C6—C5—H5	119.4	C19—C20—H20	120.4
C5—C6—C1	118.26 (13)	C20—C21—C22	120.95 (13)
C5—C6—C7	118.57 (12)	C20—C21—H21	119.5
C1—C6—C7	123.17 (13)	C22—C21—H21	119.5
C8—C7—C6	174.45 (15)	C23—C22—C21	118.62 (13)
C7—C8—C9	177.32 (15)	C23—C22—C25	121.40 (13)
C14—C9—C10	119.44 (13)	C21—C22—C25	119.97 (13)
C14—C9—C8	119.59 (13)	C24—C23—C22	121.22 (13)
C10—C9—C8	120.97 (12)	C24—C23—H23	119.4
C11—C10—C9	120.07 (14)	C22—C23—H23	119.4
C11—C10—H10	120.0	C19—C24—C23	118.81 (13)
C9—C10—H10	120.0	C19—C24—H24	120.6
C10—C11—C12	120.35 (15)	C23—C24—H24	120.6
C10—C11—H11	119.8	C22—C25—H25A	109.5
C12—C11—H11	119.8	C22—C25—H25B	109.5
C13—C12—C11	119.96 (14)	H25A—C25—H25B	109.5
C13—C12—H12	120.0	C22—C25—H25C	109.5
C11—C12—H12	120.0	H25A—C25—H25C	109.5
C12—C13—C14	120.57 (15)	H25B—C25—H25C	109.5
C12—C13—H13	119.7		
			
C6—C1—C2—C3	−1.6 (2)	O2—S1—N15—C1	34.08 (12)
N15—C1—C2—C3	178.67 (12)	C19—S1—N15—C1	−81.09 (11)
C1—C2—C3—C4	0.9 (2)	O1—S1—N15—C16	−34.38 (12)
C2—C3—C4—C5	0.7 (2)	O2—S1—N15—C16	−163.53 (10)
C3—C4—C5—C6	−1.6 (2)	C19—S1—N15—C16	81.30 (11)
C4—C5—C6—C1	1.0 (2)	C1—N15—C16—C17	−72.93 (16)
C4—C5—C6—C7	−178.24 (13)	S1—N15—C16—C17	124.65 (12)
C2—C1—C6—C5	0.64 (19)	O1—S1—C19—C24	−149.20 (11)
N15—C1—C6—C5	−179.61 (12)	O2—S1—C19—C24	−17.83 (12)
C2—C1—C6—C7	179.79 (12)	N15—S1—C19—C24	96.28 (11)
N15—C1—C6—C7	−0.4 (2)	O1—S1—C19—C20	32.94 (12)
C14—C9—C10—C11	1.5 (2)	O2—S1—C19—C20	164.32 (10)
C8—C9—C10—C11	−178.23 (13)	N15—S1—C19—C20	−81.58 (11)
C9—C10—C11—C12	−1.4 (2)	C24—C19—C20—C21	−0.3 (2)
C10—C11—C12—C13	0.2 (2)	S1—C19—C20—C21	177.52 (10)
C11—C12—C13—C14	0.9 (2)	C19—C20—C21—C22	−2.3 (2)
C12—C13—C14—C9	−0.7 (2)	C20—C21—C22—C23	3.3 (2)
C10—C9—C14—C13	−0.5 (2)	C20—C21—C22—C25	−175.95 (13)
C8—C9—C14—C13	179.28 (13)	C21—C22—C23—C24	−1.7 (2)
C2—C1—N15—C16	114.85 (14)	C25—C22—C23—C24	177.51 (13)
C6—C1—N15—C16	−64.91 (17)	C20—C19—C24—C23	1.9 (2)
C2—C1—N15—S1	−82.57 (14)	S1—C19—C24—C23	−175.95 (10)
C6—C1—N15—S1	97.67 (13)	C22—C23—C24—C19	−0.8 (2)
O1—S1—N15—C1	163.23 (10)		

### 4-Methyl-N-[2-(2-phenylethynyl)phenyl]-N-(prop-2-yn-1-yl)benzene-\ 1-sulfonamide Hydrogen-bond geometry (Å, º)

**Table:**

*D*—H···*A*	*D*—H	H···*A*	*D*···*A*	*D*—H···*A*
C20—H20···O2^i^	0.95	2.44	3.3110 (18)	152
C18—H18···O2^ii^	0.95	2.45	3.231 (2)	139

Symmetry codes: (i) −*x*+3/2, *y*−1/2, −*z*+1/2; (ii) *x*+1/2, −*y*+3/2, *z*+1/2.
